# The combined association of individuals’ psychological distress, mental health and smoking status with household expenditure

**DOI:** 10.1192/bjo.2025.10949

**Published:** 2026-03-06

**Authors:** Anita Lal, Shiva Ganjali, Nikki McCaffrey, Catherine Segan, Michelle Scollo, Mohammadreza Mohebbi

**Affiliations:** Deakin Health Economics, Institute for Health Transformation, Faculty of Health, Deakin University, Geelong, Australia; IMPACT—the Institute for Mental and Physical Health and Clinical Translation, Deakin University, Geelong, Australia; Cancer Council Victoria, Melbourne, Australia; Centre for Health Policy, School of Population and Global Health, University of Melbourne, Melbourne, Australia; Faculty of Health, Deakin University, Geelong, Australia

**Keywords:** Psychological distress, mental health, smoking, smoking cessation, household expenditure

## Abstract

**Background:**

Adults with mental illness have higher smoking prevalence and face greater financial burdens from smoking compared with the general population.

**Aims:**

This study explores how individuals’ psychological distress and smoking status are jointly associated with household expenditure patterns in Australia.

**Method:**

Daily smokers and ex-smokers were compared using the Household, Income and Labour Dynamics in Australia Survey over three waves. Psychological distress was assessed with the Kessler Psychological Distress Scale (K10) and the mental health domain of Medical Outcomes Study Short-Form General Health Survey (SF-36 MHD). Household expenditure categories included alcohol, clothing, education, fuel, general insurance, medicines, health practitioners, groceries, meals eaten out, internet, utilities, public transport and rent. Regression models using the generalised estimating equation technique compared expenditure data, controlling for age, gender, household composition, socioeconomic position, education level and wave of data collection.

**Results:**

Smokers and ex-smokers showed significant differences in expenditure across K10 psychological distress levels. At low and moderate distress levels, smokers spent more on alcohol and rent and less on insurance, health practitioners, meals out and medicines. At high distress levels, only education expenditure was significantly lower for smokers. Across SF-36 MHD tertiles, smokers spent less on education, insurance and medicine, but more on alcohol, especially at lower and moderate distress levels.

**Conclusions:**

Smoking cessation for those with moderate psychological distress may be associated with a reallocation of spending, benefiting both households and their local communities. Targeted interventions addressing smoking cessation and mental health are crucial for reducing financial and health inequities.

Smoking is the single biggest preventable cause of disability and mortality worldwide, accounting annually for 6 million deaths.^1^ By 2020, approximately 10% of deaths in Australians aged 30 years and over were attributed to smoking,^2^ so attempts to decrease smoking rates have been a public health priority. Recently, notable progress in tobacco control and substantial decreases in smoking prevalence have been observed in many countries.^3^ In Australia, where there are advanced tobacco control policies such as mass media campaigns, smoke-free environments, subsidies for pharmacological cessation aids, regulation of marketing, pictorial health warnings and world-first standardisation of tobacco product package design, the smoking prevalence has been declining steadily over the past few decades.^4^

Tobacco control policies and cessation interventions have primarily targeted the general population in Australia, with less specific attention to people with mental illness.^5^ In 2022–2023, the proportion of people experiencing high or very high levels of psychological distress increased to 24% (up from 21.0% in 2019) in Australia.^6^ The Australian Institute of Health and Welfare reported that daily smoking is up to twice as prevalent in people with mental illness compared with those without, as well as in people with high or very high levels of psychological distress compared with those with low levels of psychological distress.^6^ People with mental illness in Australia smoke more heavily and are more nicotine dependent; thus, they are more likely to experience more severe symptoms of nicotine withdrawal, some of which are difficult to distinguish from psychiatric symptoms such as depression, anxiety and anger/irritability.^7^ Despite this, people with mental illness are just as likely to make quit attempts and are more likely to use cessation supports, but are more likely to report failed quit attempts, reflective of the increased task difficulty.^8^ A combination of medication and psychological support is recommended for adults with significant levels of psychological distress to increase the chance of cessation.^9^ A Cochrane review on smoking cessation for improving mental health concludes that mental health does not worsen because of quitting smoking, and may improve with smoking cessation.^10^

As well as the considerable health burden, smoking results in significant financial burdens to individuals, their families and society.^11^ Purchasing tobacco displaces household spending from essential needs such as food, housing and clothing.^12^ As a result, households will likely experience elevated financial strain, which can be a stressor and a risk factor for more smoking as a coping mechanism to manage or alleviate stress, particularly in individuals with psychological distress.^13^ Higher intake of alcohol attributable to the comorbidity of alcohol and tobacco use,^14^ as well as healthcare costs for treating smoking-related physical illness, are other spendings that change the expenditure pattern of smoking households.^15^ Smoking cessation, by contrast, has been demonstrated to change expenditure patterns and decrease financial stress of households. Previous research showed that smoking cessation in different socioeconomic groups across Australia resulted in reallocation of spending from tobacco to less alcohol and more meals eaten out, groceries, education, motor vehicle fuel, insurance, medicines and health practitioners, leading to beneficial effects on households and local community.^16^

To the best of our knowledge, the expenditure patterns of smokers compared with ex-smokers, particularly among individuals with psychological distress, have not been previously reported. Given the high prevalence of smoking among people with mental illness, we aimed to (a) explore, using quantitative data, whether individuals’ psychological distress is differentially associated with household expenditure patterns among households with at least one current smoker compared with ex-smokers in Australia; and (b) determine whether smoking cessation is associated with reallocating household expenditure in ways that benefit both health outcomes and the broader economy.

## Method

### Data source and participants

Data were drawn from participants responding in 2013, 2015 and 2017 (waves 13 (T1), 15 (T2) and 17 (T3)) of the Household, Income, and Labour Dynamics in Australia (HILDA) Survey, a nationally representative longitudinal study initiated in 2001 to collect detailed information on the economic, social and health conditions of Australian households. At baseline (2001), the survey included 7682 households and 13 969 individuals,^17^ with annual follow-ups to track the same individuals and households over time. The survey employs a stratified multi-stage sampling design, ensuring national representativeness across diverse demographic groups in Australia. The HILDA Survey collects data through face-to-face interviews and self-completed questionnaires, providing a comprehensive understanding of many aspects of life in Australia, including household and family relationships, income and employment, and health and education. For this study, subsamples of households with at least one daily smoker or ex-smokers (individuals who reported no longer smoking) in each wave (T1, T2 and T3) were used. Participants could transition between smoking categories (e.g. moving from smoker to ex-smoker) across waves, allowing for a dynamic analysis of how changes in individual smoking status were associated with corresponding household expenditure patterns. Further details of the study design are described elsewhere.^18,19^

### Outcome variables

Annual household expenditure (AU$) on alcohol, clothing, education, fuel, health insurance, medicines, health practitioners, groceries, meals eaten out, internet, utilities, public transport and rent were derived from three waves (T1, T2 and T3), and considered as dependent variables. These are continuous variables and those with missing values were excluded from the analysis. Expenditure was adjusted to 2017 dollars by using the Australian Institute of Health and Welfare Health price inflators for healthcare-related expenditure,^20^ and the Consumer Price Index from the Australian Bureau of Statistics.^21^

### Exposure variables

#### Smoking status (smokers and ex-smokers)

The expenditure categories were compared between smokers (who self-reported smoking tobacco products (including cigarettes, cigars and pipes) daily, at the time of the survey), and ex-smokers (who previously smoked, but had since quit), with smoking status being the independent variable. Adults who had never smoked or were not daily smokers were not included, as daily smoking reflects more consistent tobacco use and expenditure patterns compared with occasional smoking.

#### Mental health domains

Mental health status was measured with the Kessler Psychological Distress Scale (K10) as the primary exposure of interest.^22^ Additionally, the mental health domain (MHD) of the Medical Outcomes Study Short-Form General Health Survey (SF-36 MHD) was used as a secondary measure of mental health status.^23^ The K10 is a simple self-reported questionnaire consisting of ten questions that ask respondents to indicate how often they have experienced specific symptoms of psychological distress, such as feeling tired for no good reason, nervous, hopeless, restless or so sad that nothing could cheer them up. The scores for the ten items are summed to produce a total score ranging from 10 to 50, which is categorised into the following levels of psychological distress: (a) low or no psychological distress (scores of 10–15), (b) moderate psychological distress (scores of 16–21), (c) high psychological distress (scores of 22–29) and (d) very high psychological distress (scores of 30-50) psychological distress levels.^24,25^ The K10 is a reliable and valid tool for assessing psychological distress in various demographic groups within Australia.^22^ The SF-36 is a widely recognised health survey designed to assess health-related quality of life. The survey comprises 36 questions spanning eight health domains: physical functioning, role limitations owing to physical difficulties (role-physical), bodily pain, general health perceptions, vitality, social functioning, role limitations owing to emotional difficulties (role-emotional) and mental health. This comprehensive survey provides detailed scores for each domain, along with summary measures for physical and mental health.^26^ Validation studies confirm the SF-36’s reliability and validity in the Australian context.^23,27^ The validity of the SF-36 data collected during the first wave of the HILDA Survey also supports its use in research examining health inequalities and population health characteristics in Australia.^23^ In this study, the MHD of the SF-36 was used, which measures aspects of psychological well-being and distress, providing a focused assessment of mental health-related quality of life. This is a standardised scale ranging from 0 to 100 with a median of 50. For the purposes of this analysis, participants’ SF-36 MHD scores were categorised into tertiles, derived from the empirical distribution of scores. The first tertile (lowest scores: 0.0–72.0) represents the worst psychological distress or poorest mental health, the second tertile (middle scores: 73.0–84.0) represents moderate psychological well-being or distress, and the third tertile (highest scores: 85.0–100.0) represents the best psychological well-being or the least distress. Several studies have employed tertiles of SF-36 scores to stratify populations by quality of life, to enhance the interpretation of differences in health outcomes.^28–30^

### Potential confounders

Several potential confounding variables commonly considered in studies on smoking and psychological distress were evaluated with covariate adjustment.^31^ These variables include age (a continuous variable), gender (a categorical variable with male and female categories), household type based on composition (including couple families with children, couple families without dependent children, single parents with children and lone persons), socioeconomic position (measured by the Socio-Economic Indexes for Areas (SEIFA) as a categorical variable in quintiles) and education levels (a categorical variable). SEIFA, developed by the Australian Bureau of Statistics, ranks areas in Australia based on relative socioeconomic advantage and disadvantage, categorising individuals by their residential areas according to factors such as income, educational attainment and occupational skills. These are indicated by qualification levels and skill categories as per the Australian and New Zealand Standard Classification of Occupations, along with the unemployment rate. SEIFA utilises data from the Australian Census of Population and Housing, conducted every 5 years, representing Census Collection Districts. So, the socioeconomic classification used in our models captures localised, community-level characteristics, rather than individual income alone.

### Statistical analysis

Descriptive statistics, including medians with interquartile ranges (IQR) for continuous variables and percentages for categorical variables, were reported for households in wave 13 (T1). Generalised estimating equation models were employed to account for within-individual correlations across the three waves.^32^ The generalised estimating equation models were specified with a Gaussian family and an identity link function, using an unstructured covariance matrix to model intra-individual correlations.^16^ The Huber–White robust variance estimator was used to ensure accurate estimation of standard errors.^33^ To avoid colinearity, separate models were utilised for K10 strata and SF-36 MHD tertiles. The independent variables included in the model were smoking status, psychological distress levels (low, moderate, high and very high, based on K10 or SF-36 MHD tertiles), socioeconomic position, index of economic resources, index of education and occupation, age, gender, household type and survey wave (waves 13, 15 and 17). Interaction terms were tested to evaluate the two-way effects of smoking status and psychological distress on household expenditure. Significant interactions were tested with Wald tests. Marginal effects and contrasts of predictive margins were computed to interpret significant interaction effects. Statistical significance was set at *p* ≤ 0.05 for main effects. To control for multiple comparisons within-groups (smoker versus ex-smoker), Bonferroni correction was applied, adjusting the significance threshold to *p* ≤ 0.01 for *post hoc* comparisons.

### Ethics approval and consent to participate

The authors assert that all procedures contributing to this work comply with the ethical standards of the relevant national and institutional committees on human experimentation and with the Helsinki Declaration of 1975, as revised in 2013. This paper uses unit record data from the HILDA Survey. The Melbourne Institute: Applied Economic and Social Research at the University of Melbourne are responsible for the design and management of the survey and ethics approval was obtained from the Office of Research Ethics and Integrity, University of Melbourne, to conduct the HILDA Survey. All experimental protocols were approved by Office of Research Ethics and Integrity, University of Melbourne. Informed consent was obtained from all participants and/or their legal guardian(s). All methods were carried out in accordance with relevant guidelines and regulations.

## Results

### Participant characteristics

The percentage of smokers varied across each wave, ranging from 22.6 to 24.3%, whereas the proportion of ex-smokers ranged from 75.7 to 77.4% (Table 1). The total numbers fluctuate slightly in each wave because of factors such as new entrants joining or leaving a continuing sample member household, deaths, or moving overseas (see Table 1). Note that the numbers may not sum to 100% because of rounding.

**Table 1:** Number of participants in each wave

Year	Daily smokers, *n* (%)	Ex-smokers, *n* (%)	Study sample total, *n*
2013 (T1)	666 (26.4)	1860 (73.6)	2526
2015	607 (24.3)	1892 (75.7)	2499
2017	579 (23.2)	1915 (76.8)	2494

Table 2 details the participant demographics at T1 (wave 13). At T1, a total of 2526 participants were analysed. Among them, 26% (*n* = 666) were smokers. Females comprised 49.1% of smokers and 50.7% of ex-smokers. Smokers tended to be younger, with a median age of 49 years (IQR: 39.0–56.0) compared with ex-smokers, who had a median age of 56 years (IQR: 46.0–66.0). Smokers had lower education levels and lower SEIFA quintiles, indicating a higher level of socioeconomic disadvantage among smokers. A higher proportion of ex-smokers had completed higher levels of education, such as postgraduate degrees (4.5%) and Bachelor’s or honours degrees (11.8%), compared with smokers (1.1 and 6.5%, respectively). Smokers also had around $12 000 lower annual incomes compared with ex-smokers. Geographically, more smokers resided in regional than metropolitan areas (47.6 *v*. 38.8%), whereas more ex-smokers were in major cities (59.8 *v*. 50.6%).

**Table 2:** Participants demographics in wave 13 (T1) for cigarette-only smokers

Characteristics	T1 (Wave 13)		
	Smokers (*n* = 666)	Ex-smokers (*n* = 1860)	*p*-value
Gender			
Women (%)	49.1	50.7	0.47
Men (%)	50.9	49.3	
Age, years, median (IQR)	49.0 (39.0–56.0)	56.0 (46.0–66.0)	<0.001
K10 scales			
Low (%)	57.5	69.4	<0.001
Moderate (%)	18.9	19.1	
High (%)	13.7	8.4	
Very high (%)	9.9	3.1	
SF-36 MHD tertiles			
Tertile 1 (%)	46.5	36.1	<0.001
Tertile 2 (%)	29.5	30.4	
Tertile 3 (%)	24.0	33.5	
Household type			
Couple family with children or others (%)	52.3	69.2	<0.001
Couple family with no children (%)	5.7	6.5	
Single parent with children (%)	13.2	5.8	
Lone person (%)	28.7	18.5	
Education			
Undetermined (%)	0.0	0.1	<0.001
Year 11 or below (%)	37.6	30.4	
Year 12 (%)	12.3	10.7	
Certificate III or IV (%)	29.5	25.5	
Advanced diploma, diploma (%)	8.9	9.8	
Bachelor or honours (%)	6.5	11.8	
Graduate diploma or certificate (%)	4.2	7.1	
Postgraduate – Master’s or doctorate (%)	1.1	4.5	
State of residence			
New South Wales (%)	25.6	27.9	<0.001
Victoria (%)	21.6	23.7	
Queensland (%)	25.2	22.3	
South Australia (%)	11.5	9.6	
Western Australia (%)	7.8	10.4	
Tasmania (%)	6.8	3.4	
Northern Territory (%)	0.8	0.5	
Australian Capital Territory (%)	0.8	22.2	
Remoteness of area			
Major city (%)	50.6	59.8	<0.001
Inner regional (%)	33.2	28.1	
Outer regional (%)	14.4	10.7	
Remote and very remote (%)	1.7	1.4	
Household financial year gross total income (AU$), median (IQR)	$75 037.0 ($40 230.0–$119 995.5)	$87 250.0 ($43 801.0–$143 005.0)	<0.001
Decile of index of socioeconomic advantage (SEIFA), median (IQR)	4.0 (2.0–6.5)	6.0 (3.0–8.0)	<0.001
Decile of index of economic resources, median (IQR)	5.5 (2.0–7.0)	6.0 (3.0–8.0)	<0.001
Decile of index of education and occupation, median (IQR)	5.5 (2.0–7.0)	6.0 (3.0–8.0)	<0.001

IQR, Interquartile range; K10, Kessler Psychological Distress Scale; SF-36 MHD, mental health domain of the 36-item Short-Form Health Survey; SEIFA, Socioeconomic Index for Areas.

The percentages of individuals experiencing high (13.7 *v*. 8.4%) and very high (9.9 *v*. 3.1%) psychological distress based on the K10 scale was higher among current smokers than among ex-smokers. Additionally, the percentage of individuals reporting worse mental health status based on the SF-36 MHD questionnaire was also higher for current smokers than for ex-smokers (46.5 *v*. 36.1%), whereas ex-smokers had a higher percentage than current smokers in the highest tertile of the SF-36 MHD, indicating better mental health status (33.5 *v*. 24.0%).

### Household expenditure

The results for adjusted mean expenditure on various household items based on psychological distress levels, measured by both K10 scales and the SF-36 MHD, are summarised in Table 3. The results for K10 strata indicate that individuals with very high levels of psychological distress spent significantly less on alcohol (−$342.3, 95% CI −$571.3 to −$113.3), fuel (−$596.0, 95% CI −$810.3 to −$25.4), general insurance (−$400.2, 95% CI −$539.9 to −$260.5), groceries (−$788.4, 95% CI −$1287.4 to −$289.3), meals out (−$549.7, 95% CI −$47.4 to −$34.8) and utilities (−$318.5, 95% CI −$490.5 to −$146.5) compared with those with low psychological distress level. The findings suggest as psychological distress levels increase, there is a general decrease in expenditure on these items. Conversely, individuals with very high levels of psychological distress spent significantly more on medicine (+$111.0, 95% CI $34.1−$187.9) and rent (+$78.7, 95% CI $13.9−$143.6) compared with those with the lower levels of psychological distress, with a general increase as psychological distress levels increase. In addition, different tertiles of SF-36 MHD showed a significant effect of the expenditure on fuel, insurance, meals out and medicine. Specifically, the results demonstrate individuals in tertile 1 of the SF-36 MHD (worst mental health) spent significantly less on fuel (−$175.4, 95% CI −$295.6 to −$55.2,), insurance (−$118.3, 95% CI −$189.8 to −$46.8) and meals out (−$193.6, 95% CI −$302.5 to −$84.7), with decreasing expenditure on these items as psychological well-being worsened. Mean expenditure on medicines significantly increased as psychological well-being worsened (tertiles 1 and 2).

**Table 3:** Adjusted mean expenditure (AU$) on various household items by K10 and SF-36 MHD levels

Household item	K10 scales Coefficient (95% CI)					SF-36 MHD Coefficient (95% CI)			
	Low	Moderate	High	Very high	*p*-value^a^	Tertile 3	Tertile 2	Tertile 1 (worst mental health)	*p*-value^a^
Alcohol	As reference	−49.3 (−175.9 to 77.3)	−196.0^*^ (−374.5 to −17.5)	−342.3** (−571.3 to −113.3)	0.016	As reference	−35.8 (−101.9–30.2)	8.1 (−70.3 to 86.6)	0.324
Clothing		103.3 (−97.9 to 304.6)	−55.5 (−22.8 to 111.7)	−309.6^*^ (−582.0 to −37.2)	0.173		62.1 (−31.8 to 156.1)	77.0 (−36.2 to 190.2)	0.236
Education		−201.4 (−499.1 to 96.4)	−123.5 (−441.0 to 194.1)	−255.3 (−544.9 to 34.4)	0.335		−30.3 (−154.5 to 94.0)	−5.6 (−133.2 to 144.3)	0.867
Fuel		−67.0 (−202.7 to 68.7)	−212.7 (−457.0 to 31.5)	−596.0*** (−810.3 to −25.4)	<0.001		−88.7 (−201.9 to 24.5)	−175.4** (−295.6 to −55.2)	<0.05
General insurance		−154.8** (−252.4 to −57.1)	−239.2 *** (−370.4 to −108.1)	−400.2*** (−539.9 to −260.5)	<0.001		50.1^*^ (−113.8 to 13.6)	−118.3** (−189.8 to −46.8)	<0.01
Groceries		−229.7 (−509.5 to 50.2)	−116.2 (−566.5 to 334.1)	−788.4** (−1287.4 to −289.3)	<0.05		64.7 (−105.0 to 234.4)	−18.4 (−207.7 to 171.0,)	0.538
Health practitioner		154.3^*^ (13.6–295.0)	289.0 (−59.9 to 637.8)	−24.8 (−275.9 to 223.3)	0.076		58.9 (−23.2 to 141.0)	60.5 (−31.0 to 152.0)	0.288
Internet		−101.5 (−238.7 to 35.7)	−172.0 (−355.6 to 11.7)	−111.0 (−320.6 to 98.6)	0.141		0.6 (−77.2 to 78.3)	−27.9 (−111.3 to 55.4)	0.740
Meals out		−82.0 (−255.6 to 91.5)	−173.4 (−419.5 to 72.7)	−549.7*** ^*^(−47.4 to −34.8)	<0.001		−104.7^*^ (−202.9 to −6.6)	−193.6*** (−302.5 to −84.7)	<0.01
Medicines		8.3 (−46.1 to 62.7)	74.9^*^ (14.6 to 135.3)	111.0** (34.1–187.9)	<0.01		13.9^*^ (−24.1 to 49.8)	44.5^*^ (10.7–78.2)	<0.05
Public transport		8.9 (−92.7 to 110.5)	42.4 (−57.0 to 141.9)	−16.6 (−115.5 to 82.2)	0.722		11.1 (−27.5 to 21.4)	35.1 (−9.0 to 79.2)	0.295
Rent		31.2^*^ (4.9–57.4)	29.9 (−5.9 to 65.7)	78.7^*^ (13.9–143.6)	<0.05		5.0 (−11.3 to 21.2)	17.3 (−0.5 to 35.1)	0.123
Utilities		−39.0 (−158.8 to 80.9)	−41.1 (−167.6 to 85.5)	−318.5*** (−490.5 to −146.5)	<0.01		−46.4 (−129.5 to 36.7)	−48.2 (−127.7 to 31.3)	0.460

K10, Kessler Psychological Distress Scale; SF-36 MHD, mental health domain of the 36-item Short-Form Health Survey.

Adjusted for smoking, waves, age, gender, education/occupation, index of economic resources, household type, socioeconomic advantage and disadvantage (Socioeconomic Index for Areas). Bold font indicates significance in comparison to the reference group.

a Overall *p*-value of K10 for adjusted model.

* *p* < 0.05, **p < 0.01, ***p < 0.001.

Furthermore, household type-specific effects were tested by employing additional models that included interactions between household type, smoking status and K10. However, the results did not indicate any significant modification effect of household type on the relationship between smoking status and psychological distress with respect to expenditure items.

Table 4 compares the difference in expenditure of smokers and ex-smokers at each level of psychological distress based on the K10 and SF-36 MHD. The mean expenditure on alcohol, clothing, education, insurance, health practitioners, meals out, medicines and rent were significantly different between smokers and ex-smokers at different levels of K10; specifically, at lower levels, smokers spent significantly more on alcohol and rent, and less on clothing, education, fuel, general insurance, groceries, health practitioners, meals out and medicine than ex-smokers. Smokers with a moderate level of psychological distress spent significantly more on alcohol and rent, and less on general insurance, health practitioners, meals out and medicines, compared with ex-smokers. At high and very high levels of the K10, the only significant difference in expenditure was on education for those with high levels of distress, which was significantly lower (−$490.4) for smokers than for ex-smokers. The results for smoking any tobacco product (Supplementary Table 1 available at https://doi.org/10.1192/bjo.2025.10949) were similar to those for cigarette smoking only (Table 3).

**Table 4:** Joint effect of smoking status (cigarette-only smokers versus ex-smokers) and psychological distress (K10) and mental health (SF-36 MHD) on mean expenditure (AU$) per annum

Household item	K10 scales Contrast (95% CI)					SF-36 MHD tertiles Contrast (95% CI)			
	Low	Moderate	High	Very high	Joint *p*-value	Tertile 3	Tertile 2	Tertile 1 (worst mental health)	Joint *p*-value
Alcohol	300.2^*^ (66.3–534.1)	364.3^*^ (43.5–685.1)	179.5 (−176.7 to 535.8)	−80.8 (−498.0 to 336.4)	<0.05	177.5 (−16.5 to 371.5)	178.3^*^ (9.1–347.5)	234.8** (66.0–403.6)	<0.05
Clothing	−384.9*** (−522.0 to −247.9)	−114.10 (−613.9 to 385.7)	−103.10 (−371.98 to 165.79)	227.73 (−244.4 to 699.9)	<0.001	−237.1** (−413.4 to −60.9)	−356.0^*^ (−$653.8 to −$58.2)	60.4 (−440.2 to 561.0)	<0.05
Education	−507.1** (−825.8 to −188.3)	−31.4 (−439.0 to $376.2)	−$490.4^*^ (−958.1 to −22.7)	−189.6 (−576.8 to 197.6)	<0.01	−226.7^*^ (−504.5 to 51.1)	−222.3^*^ (−$429.6 to −$14.9)	−491.3*** (−678.6 to −303.9)	<0.001
Fuel	−275.4^*^ (−517.0 to −33.9)	44.6 (−250.4 to 339.7)	15.9 (−692.8 to 724.6)	−277.4 (−706.3 to 151.4)	0.118	−243.1^*^ (−447.6 to −38.6)	−117.7 (−331.9 to 96.4)	−94.2 (−320.8 to 132.4)	0.130
General insurance	−380.7*** (−537.0 to −224.3)	−395.4*** (−576.5 to −214.2)	−220.9 (−418.1 to 69.1)	−190.6 (−418.1 to 37.0)	<0.001	−289.2*** (−423.7 to −154.8)	−331.0*** (−453.0 to −209.1)	−216.1*** (−336.2 to −96.0)	<0.001
Groceries	−478.2^*^ (−928.8 to −27.6)	−226.0 (−808.8 to 356.8)	−558.7 (−1356.4 to 239.1)	−389.9 (−1270.2 to 490.4)	0.174	−994.3*** (−1380.2 to −608.3)	−256.5 (−650.4 to 137.5)	−339.2^*^ (−656.0 to −22.5)	<0.001
Health practitioners	−323.8*** (−451.3 to −196.3)	−392.0** (−628.1 to −155.9)	−$380.3 (−974.5 to 232.0)	−334.7 (−708.1 to 38.7)	<0.001	−18.7 (−192.7 to 155.3)	−64.1 (−205.3 to 77.1)	−31.4 (−176.6 to 113.9)	0.833
Internet	48.2 (−136.2 to 232.7)	145.1 (−87.6 to 377.7)	−173.2 (−495.8 to 149.4)	−94.2 (−425.6 to 237.3)	0.501	−18.7 (−192.7 to 155.3)	−64.1 (−205.3 to 77.1)	−31.4 (−176.6 to 113.9)	0.833
Meals out	−370.6^*^ (−622.0 to −119.1)	−500.1** (−813.1 to −187.1)	−211.6 (−619.9 to 196.7)	−345.8 (−759.1 to 67.6)	<0.01	−504.6*** (−713.2 to −296.0)	−196.0 (−418.7 to 26.7)	−326.4*** (−485.3 to −167.4)	<0.001
Medicines	−$108.9*** (−$165.7 to −$52.1)	−$123.8** (−$202.7 to −$44.9)	−$8.7 (−$129.9 to $112.5)	−$7.3 (−$168.5 to $153.9)	<0.001	−112.8*** (−169.4 to −56.2)	−58.7^*^ (−108.2 to −9.1)	−56.0^*^ (−101.0 to −10.9)	<0.001
Public transport	−$46.4 (−$127.4 to $34.6)	−$21.4 (−$183.5 to $140.7)	−$124.9 (−$288.3 to $38.6)	$65.7 (−$92.9 to $224.3)	0.304	−57.1 (−135.0 to 20.8)	−$79.8^*^ (−$141.7 to −$17.8)	−$58.8 (−$118.2 to $0.6)	<0.05
Rent	59.6^*^ ($12.4–$106.8)	71.2^*^ (10.2–132.1)	−23.3 (−93.3 to 46.7)	−110.6 (−229.9 to 8.6)	<0.05	57.1** (21.8–92.4)	36.4^*^ (0.6–72.2)	15.1 (−14.0 to 4.1)	<0.05
Utilities	−86.9 (−239.4 to 65.6)	−20.0 (−238.5 to 198.5)	−16.1 (−259.2 to 226.9)	−245.0 (−579.0 to 88.9)	0.510	41.2 (−142.4 to 224.9)	3.9 (−130.3 to 138.1)	−60.2 (−175.6 to 55.2)	0.621

K10, Kessler Psychological Distress Scale; SF-36 MHD, mental health domain of 36-item Short-Form Health Survey. Bold font indicates significance.

* *p* < 0.05,**p < 0.01,***p < 0.001.

Smokers in all tertiles of the SF-36 MHD, spent significantly less than ex-smokers on education (ranging from −$491.3 in tertile 1 to −$226.7 in tertile 3), general insurance (ranging from −$216.1 in tertile 1 to −$289.2 in tertile 3) and medicine (ranging from −$56.0 in tertile 1 to −$112.8 in tertile 3). Smokers in tertiles 1 and 3 of the SF-36 MHD spent significantly less on meals out (−$326.4 and −$504.6, respectively) than ex-smokers. In contrast, the mean expenditure on alcohol was significantly higher in smokers in tertiles 1 and 2 of the SF-36 MHD (+$234.8 and +178.3, respectively) than ex-smokers.

Annual tobacco-related expenditure among smokers increased over time across survey waves, ranging from $3947.03 in wave 13 (T1) to $4826.62 in wave 17 (T3). This upward trend was observed across all psychological distress levels. However, a general decline in tobacco-related spending was evident with increasing levels of psychological distress, with individuals reporting moderate and high distress spending less than those with low distress. The mean annual expenditure at T1 for different spending categories by K10 strata in each smoker and ex-smoker group, is shown in Supplementary Table 2. In all categories of psychological distress, smokers tended to spend more on alcohol and rent, and less on clothing, education, general insurance, groceries, health practitioners, meals out and medicines and utilities compared with ex-smokers. As the severity of psychological distress increased, the expenditure on education, health practitioners, general insurance, meals out, medicines and utilities were reduced considerably for smokers. There were no notable differences in expenditure for internet, fuel and public transport between smokers and ex-smokers. Supplementary Fig. 1 depicts the mean annual household expenditure categories across waves by psychological distress levels (K10) among smokers and ex-smokers.

## Discussion

This study found that the spending habits of smokers and ex-smokers differ significantly, and there is evidence that this could be shaped by their levels of psychological distress (K10). At low and moderate levels of psychological distress, smokers spend significantly more than ex-smokers on alcohol and rent, and significantly less than ex-smokers on health practitioners, meals out, medicines and general insurance. At low levels of psychological distress, smokers spent less on clothing, groceries and fuel. The spending disparities between smokers and ex-smokers diminish at high and very high levels of psychological distress, except for education where at all levels, smokers spent less. These spending patterns demonstrate how smoking and psychological distress can worsen access to health services and opportunities for personal growth. The results also reinforce the persistent tobacco-related inequality faced by individuals with psychological distress.

The financial burden associated with smoking in those with mental illness is profound. Individuals with mental illness not only bear the regular costs of purchasing tobacco products, but also face the financial challenges of managing mental health conditions. Smokers with a mental illness spend up to 30–40% of their income on cigarettes, affecting their ability to afford necessities such as food and clothing.^34,35^ This study enhances the understanding by quantifying the specific expenditure differences across various categories. The finding that smokers consistently spend less on education across all levels of distress aligns with previous research using the HILDA survey comparing smokers and ex-smokers. Overall, ex-smokers had increased spending on education.^16^ These findings also align with earlier studies that have highlighted lower household spending amongst smokers than ex-smokers in categories such as groceries and meals out, among lower socioeconomic groups in Australia.^16^

Tailored smoking cessation programmes that tackle the psychological challenges of this group could lead to significant improvements in health,^36^ as well as financial benefits resulting from shifts in household spending.^37^ Through lower spending on alcohol and increasing expenditure on health, education and groceries, smoking cessation could positively affect both individual households and their wider communities. Lowering smoking rates among individuals with high psychological distress can also lead to reduced incidence of smoking related diseases and reductions in healthcare costs for individuals, their families and their communities.^38^

These findings have important implications for public health strategies aimed at lowering smoking rates among individuals with mental illness. Currently, mental health services do not routinely offer or link to smoking cessation.^39^ There is increasing evidence that smoking can negatively affect mental health because of the tobacco withdrawal cycle and, conversely, quitting smoking is likely to enhance mental health.^40,41^ However, the Clinician Guidelines for Psychiatrists in Australia and New Zealand state ‘The management of tobacco smoking is one of the most important activities a mental health clinician or service can undertake in terms of reducing mortality, improving quality of life and improving efficacy of mental health treatment.^’42^ Innovative approaches should be considered to address the combined challenges faced by the population with psychological distress and mental illness.^43,44^

Multi-session cessation services, such as the Quitline in Australia, which is effective and cost-effective, have tailored protocols for people with mental illness.^45–47^ People with mental illness are provided with extra sessions, psychoeducation on the mental health benefits of smoking cessation and monitoring of mood and nicotine withdrawal symptoms. Counselling incorporates mood management strategies that can dually act as cessation strategies such as relaxation, exercise and scheduling pleasant activities. People are strongly encouraged to contact their doctor for cessation medicines, for medication review and to help monitor mental health symptoms. Providing comprehensive and cost-effective support that includes counselling, mental health services and stress management techniques could improve the effectiveness of smoking cessation efforts.^48^ Making sure these programmes are easily accessible to those in need, including through community health centres and online platforms, is essential for reducing health disparities.

### Strengths and limitations

This study leverages the strengths of the HILDA Survey, including the large, nationally representative longitudinal sample of smokers and ex-smokers, allowing for comprehensive analysis over various waves and multiple mental health measures. The findings of this study should be considered in light of several limitations. Since the HILDA survey is conducted annually, precise measures of quitting at shorter intervals are lacking. Data may also be prone to reporting biases. Two mental health measures, the K10 and the SF-36 MHD have been reported to help mitigate bias. We did not include physical health conditions, physical activity, housing tenure or other lifestyle factors such as diet, social engagement or social isolation as potential confounders. Those in the private rental market would face considerably more financial stress than those on equivalent income in social housing, including public housing, where rents are set at a fixed percentage of income. The presence of chronic health conditions could also affect the utilisation of healthcare and medicines. And finally, because of the observational study design and the possibility of residual confounding and biases inherent to survey data, only associations and co-occurrence can be concluded from this study, and causal inference is limited.

In conclusion, this study highlights the significant financial burden associated with smoking and psychological distress on household expenditure. For individuals experiencing psychological distress, quitting smoking is associated with more favourable spending patterns, which may reflect potential benefits for individuals, their households and their communities. Targeted interventions that address both smoking cessation and mental health are crucial for reducing inequalities of the financial burden of smoking and for reducing health inequities.

## Supporting information

Lal et al. supplementary material 1 Lal et al. supplementary material

Lal et al. supplementary material 2 Lal et al. supplementary material

## Data Availability

The survey data that support the findings of this study are available in Australian Data Archive Dataverse with the identifier https://dataverse.ada.edu.au/dataset.xhtml?persistentId=doi:10.26193/BBOTSM

## Supplementary material

The supplementary material is available online at https://doi.org/10.1192/bjo.2025.10949

## References

[1] Levy DT , Levy J , Mauer-Stender K. Potential impact of strong tobacco-control policies in 11 newly independent states. Central Eur J Public Health 2019; 27: 115–26. 10.21101/cejph.a5506 31241286

[2] Dai X , Gakidou E , Lopez AD. Evolution of the global smoking epidemic over the past half century: strengthening the evidence base for policy action. Tob Control 2022; 31: 129–37. 35241576 10.1136/tobaccocontrol-2021-056535

[3] Polaris Observatory Collaborators. Global prevalence, cascade of care, and prophylaxis coverage of hepatitis B in 2022: a modelling study. Lancet Gastroenterol Hepatol 2023; 8: 879–907. 37517414 10.1016/S2468-1253(23)00197-8

[4] Wilkinson AL , Scollo MM , Wakefield MA , Spittal MJ , Chaloupka FJ , Durkin SJ. Smoking prevalence following tobacco tax increases in Australia between 2001 and 2017: an interrupted time-series analysis. Lancet Public Health 2019; 4: e618–27. 31759897 10.1016/S2468-2667(19)30203-8

[5] White S , McCaffrey N , Scollo M. Tobacco dependence treatment in Australia – an untapped opportunity for reducing the smoking burden. Public Health Res Pract 2020; 30: 3032020. 36823796 10.17061/phrp3032020

[6] Australian Institute of Health and Welfare. Mental Health and Use of Alcohol, Tobacco, e–Cigarettes and Other Drugs. Australian Institute of Health and Welfare, 2024 (https://www.aihw.gov.au/reports/mental-health/mental-health-alcohol-drugs).

[7] Hughes JR. Measurement of the effects of abstinence from tobacco: a qualitative review. Psychol Addict Behav 2007; 21: 127–37. 17563132 10.1037/0893-164X.21.2.127

[8] McClave AK , McKnight-Eily LR , Davis SP , Dube SR. Smoking characteristics of adults with selected lifetime mental illnesses: results from the 2007 National Health Interview Survey. Am J Public Health 2010; 100: 2464–72. 20966369 10.2105/AJPH.2009.188136 PMC2978196

[9] Taylor GMJ , Munafò MR. What about treatment of smoking to improve survival and reduce depression? Lancet Psychiatry 2018; 5: 464. 10.1016/S2215-0366(18)30132-9 29857836

[10] Taylor GMJ , Lindson N , Farley A , Leinberger-Jabari A , Sawyer K , te Water Naudé R , et al. Smoking cessation for improving mental health. Cochrane Database Syst Rev 2021; 3: CD013522. 33687070 10.1002/14651858.CD013522.pub2 PMC8121093

[11] Prochaska JJ. Smoking and mental illness—breaking the link. New Engl J Med 2011; 365: 196–8. 21774707 10.1056/NEJMp1105248 PMC4457781

[12] Guillaumier A , Bonevski B , Paul C. ‘Cigarettes are priority’: a qualitative study of how Australian socioeconomically disadvantaged smokers respond to rising cigarette prices. Health Educ Res 2015; 30: 599–608. 26116583 10.1093/her/cyv026

[13] Widome R , Joseph AM , Hammett P , Van Ryn M , Nelson DB , Nyman JA , et al. Associations between smoking behaviors and financial stress among low-income smokers. Prev Med Rep 2015; 2: 911–5. 26844167 10.1016/j.pmedr.2015.10.011 PMC4721304

[14] Adams S. Psychopharmacology of tobacco and alcohol comorbidity: a review of current evidence. Curr Addict Rep 2017; 4: 25–34. 28357192 10.1007/s40429-017-0129-z PMC5350203

[15] Brook A , Zhang C. The role of personal attributes in the genesis and progression of lung disease and cigarette smoking. Am J Publ Health 2013; 103: 931–7. 10.2105/AJPH.2012.300748 PMC3530664 22994182

[16] Lal A , Mohebi M , White SL , Scollo M , McCaffrey N. Household expenditure of smokers and ex-smokers across socioeconomic groups: results from a large nationwide Australian longitudinal survey. BMC Public Health 2022; 22: 1706. 36076210 10.1186/s12889-022-14083-y PMC9461138

[17] Watson N , Wooden M. The HILDA survey: progress and future developments. Austr Econ Rev 2010; 43: 326–36.

[18] Department of Social Services, Melbourne Institute of Applied Economic Social Research. The Household, Income and Labour Dynamics in Australia (HILDA) Survey, RESTRICTED RELEASE 18 (Waves 1–18). ADA Dataverse, 2022 (10.26193/IYBXHM).

[19] Watson N. Finding your way around the HILDA survey data. Austr Econ Rev 2021; 54: 554–64.

[20] Australian Institute of Health and Welfare. Health Expenditure Australia 2017-18 Supplementary Tables. Australian Institute of Health and Welfare, 2019 (https://www.aihw.gov.au/reports/health-welfare-expenditure/health-expenditure-australia-2017-18/data).

[21] Australian Bureau of Statistics. Consumer Price Index Australia TABLE 7. CPI: Group, Sub-group and Expenditure Class, Weighted Average of Eight Capital Cities. Australian Bureau of Statistics, 2020 (https://www.abs.gov.au/statistics/economy/price-indexes-and-inflation/consumer-price-index-australia/dec-2020).

[22] Blake JA , Farugia TL , Andrew B , Malacova E , Lawrence D , Thomas HJ , et al. The Kessler Psychological Distress Scale in Australian adolescents: analysis of the second Australian Child and Adolescent Survey of Mental Health and Wellbeing. Aust N Z J Psychiatry 2024; 58: 345–54. 38095118 10.1177/00048674231216601

[23] Butterworth P , Crosier T. The validity of the SF-36 in an Australian National Household Survey: demonstrating the applicability of the Household Income and Labour Dynamics in Australia (HILDA) Survey to examination of health inequalities. BMC Publ Health 2004; 4: 44. 10.1186/1471-2458-4-44 PMC524495 15469617

[24] Kessler RC , Barker PR , Colpe LJ , Epstein JF , Gfroerer JC , Hiripi E , et al. Screening for serious mental illness in the general population. Arch Gen Psychiatry 2003; 60: 184–9. 12578436 10.1001/archpsyc.60.2.184

[25] Wooden M. Use of the Kessler Psychological Distress Scale in the HILDA Survey . Melbourne Institute of Applied Economic and Social Research, 2009 (https://melbourneinstitute.unimelb.edu.au/assets/documents/hilda-bibliography/hilda-discussion-papers/hdps209.pdf).

[26] Ware JE . SF-36 health survey update. Spine 2000; 25: 3130–9. 11124729 10.1097/00007632-200012150-00008

[27] McCallum J. The SF-36 in an Australian sample: validating a new, generic health status measure. Austr J Public Health 1995; 19: 160–6. 10.1111/j.1753-6405.1995.tb00367.x 7786942

[28] van Gestel YRBM , Hoeks SE , Sin DD , Stam HJ , Mertens FW , Bax JJ , et al. Beta-blockers and health-related quality of life in patients with peripheral arterial disease and COPD. I Int J Chron Obstruct Pulmon Dis 2009; 4: 177–83. 19516916 10.2147/copd.s5511 PMC2685144

[29] Tosteson ANA , Kneeland TS , Nease RF , Sumner W. Automated current health time-trade-off assessments in womens health. Value Health 2002; 5: 98–105. 11918825 10.1046/j.1524-4733.2002.52102.x

[30] Rumsfeld JS , Magid DJ , Plomondon ME , Sales AE , Grunwald GK , Every NR , et al. History of depression, angina, and quality of life after acute coronary syndromes. Am Heart J 2003; 145: 493–9. 12660673 10.1067/mhj.2003.177

[31] Elze Markus C , Gregson J , Baber U , Williamson E , Sartori S , Mehran R , et al. Comparison of propensity score methods and covariate adjustment. JACC 2017; 69: 345–57. 28104076 10.1016/j.jacc.2016.10.060

[32] Christopher JWZ. Generalized estimating equation models for correlated data: a review with applications. Am J Polit Sci 2001; 45: 470–90.

[33] Freedman DA. On the so-called ‘huber sandwich estimator’ and ‘robust standard errors’. Am Stat 2006; 60: 299–302.

[34] Brown E , O’Donoghue B , White SL , Chanen AM , Bedi G , Adams S , et al. Tobacco smoking in young people seeking treatment for mental ill-health: what are their attitudes, knowledge and behaviours towards quitting? Irish J Psychol Med 2020; 38: 30–9. 10.1017/ipm.2020.18 32317033

[35] Salt V , Osborne C. Mental health, smoking and poverty: benefits of supporting smokers to quit. BJPsych 2020; 44: 213–8. 10.1192/bjb.2020.88 PMC7525592 32847647

[36] Fibbins H , Ward PB , Morell R , Lederman O , Teasdale S , Davies K , et al. Evaluation of a smoking cessation program for adults with severe mental illness in a public mental health service. J Psychiatr Mental Health Nurs 2024; 31: 990–7. 10.1111/jpm.13052 38551076

[37] Latkin CA , Tseng TY , Davey-Rothwell M , Kennedy RD , Moran MB , Czaplicki L , et al. The relationship between neighborhood disorder, social networks, and indoor cigarette smoking among impoverished inner-city residents. J Urban Health 2017; 94: 534–41. 28560613 10.1007/s11524-017-0170-1 PMC5533668

[38] Ekpu VU , Brown AK. The economic impact of smoking and of reducing smoking prevalence: review of evidence. Tob Use Insights 2015; 8: 1–35. 26242225 10.4137/TUI.S15628 PMC4502793

[39] Greenhalgh EM , Brennan E , Segan C , Scollo M. Monitoring changes in smoking and quitting behaviours among Australians with and without mental illness over 15 years. Austr N Z J Public Health 2022; 46: 223–9. 10.1111/1753-6405.13185 34821438

[40] Taylor GMJ , Munafò MR. Does smoking cause poor mental health? Lancet Psychiatry 2019; 6: 2–3. 30527762 10.1016/S2215-0366(18)30459-0

[41] Taylor G , McNeill A , Girling A , Farley A , Lindson-Hawley N , Aveyard P. Change in mental health after smoking cessation: systematic review and meta-analysis. BMJ 2014; 348: g1151. 24524926 10.1136/bmj.g1151 PMC3923980

[42] Coleman M , Ridley K , Arunogiri S , Thornton P , Faint N . Mental Health Clinician Guidance for Managing People’s Smoking Cessation. The Royal Australian & New Zealand College of Psychiatrists, 2022 (https://www.ranzcp.org/getmedia/6ebd2e07-e683-44ac-9671-995904cd7fcb/ranzcp-guidance-for-management-of-smoking-cessation.pdf).

[43] Sheals K , Tombor I , McNeill A , Shahab L. A mixed-method systematic review and meta-analysis of mental health professionals attitudes toward smoking and smoking cessation among people with mental illnesses. Addiction 2016; 111: 1536–53. 27003925 10.1111/add.13387 PMC5025720

[44] Campion J , Johnston G , Shiers D , Chew-Graham C. Why should we prioritise smoking cessation for people with mental health conditions? Br J Gen Pract 2023; 73: 251. 37230792 10.3399/bjgp23X732921 PMC10229168

[45] Cancer Council Victoria. National Minimum Quitline Standards. Cancer Council Victoria, 2024 (https://www.cancervic.org.au/get-support/for-health-professionals/national-quitline-standards).

[46] Lal A , Mihalopoulos C , Wallace A , Vos T. The cost-effectiveness of call-back counselling for smoking cessation. Tob Control 2014; 23: 437–42. 23748188 10.1136/tobaccocontrol-2012-050907

[47] Crosland P , Scollo M , White SL , McCaffrey N. Cost-effectiveness and productivity impacts of call-back telephone counselling for smoking cessation. Public Health Res Pract 2023; 33: 33232306. 37287193 10.17061/phrp33232306

[48] Taylor GMJ , Sawyer K , Kessler D , Munafò MR , Aveyard P , Shaw A. Views about integrating smoking cessation treatment within psychological services for patients with common mental illness: a multi-perspective qualitative study. Health Expect 2021; 24: 411–20. 33368996 10.1111/hex.13182 PMC8077097

