# Supplementary material for: The combined association of individuals’ psychological distress, mental health and smoking status with household expenditure
Source: BJPsych Open. 2026 Mar 6;12(2):e81. doi: 10.1192/bjo.2025.10949 (PMC13107326; doi:10.1192/bjo.2025.10949)
Supplement: Lal et al. supplementary material 1 — Lal et al. supplementary material [file S2056472425109496sup001.pdf]

Figure S1 Adjusted prediction of household expenditure (A$) by psychological distress levels (K10) among smokers and ex-smokers

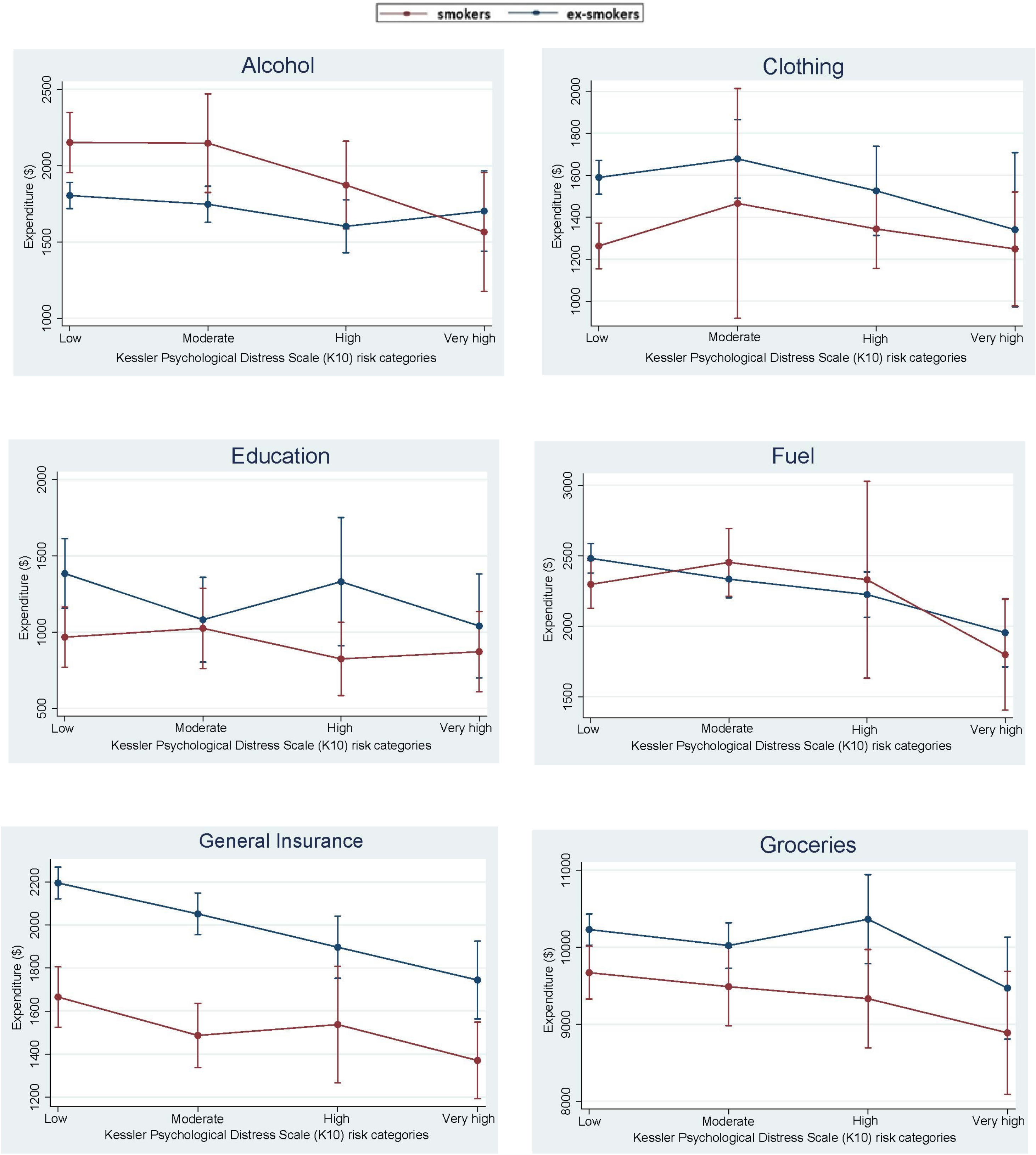

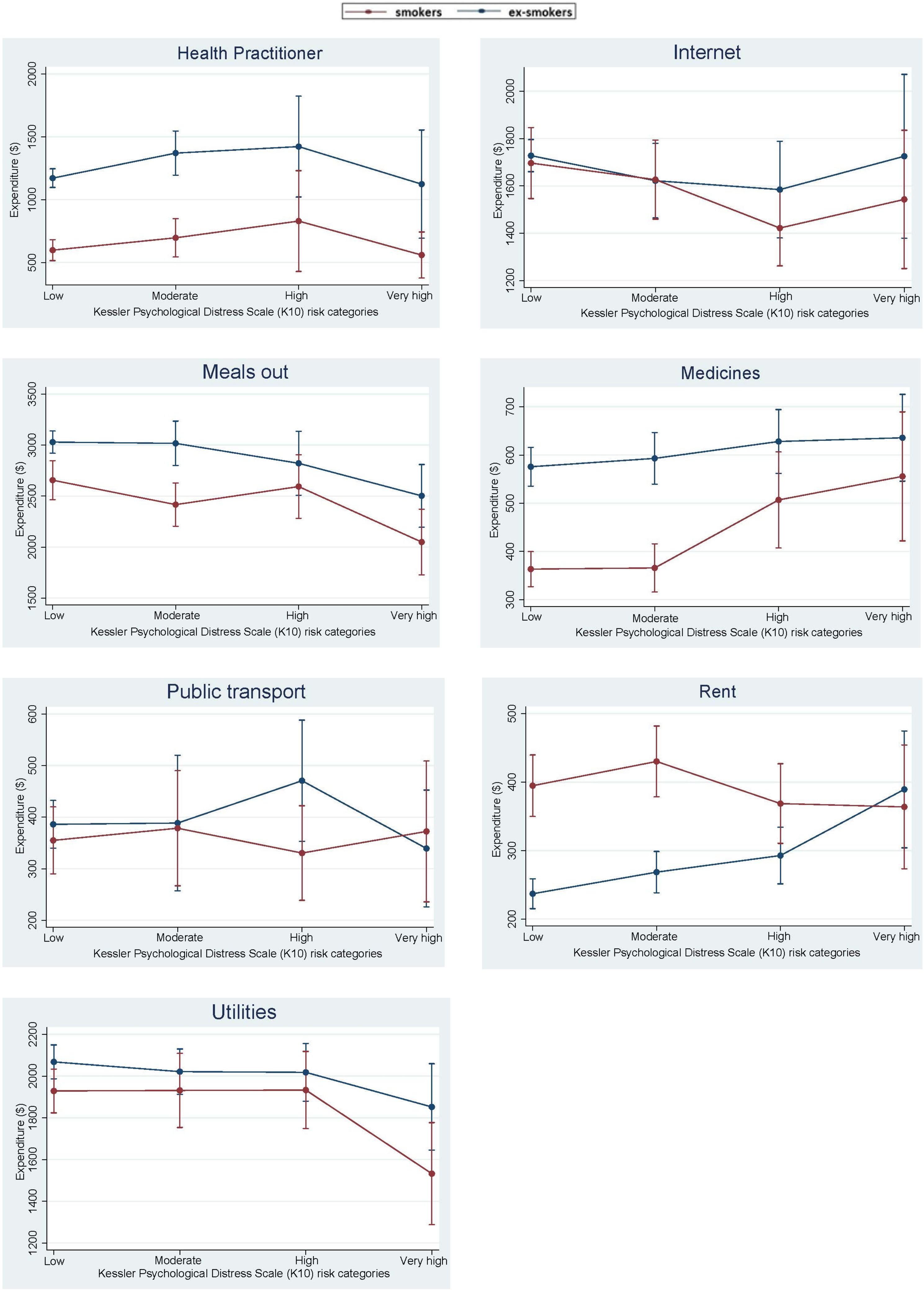
smokers
ex-smokers
Health Practitioner
Internet
Meals out
Medicines
Public transport
Rent
Utilities
Expenditure ($)
Low
Moderate
High
Very high
Kessler Psychological Distress Scale (K10) risk categories
