# Supplementary material for: The combined association of individuals’ psychological distress, mental health and smoking status with household expenditure
Source: BJPsych Open. 2026 Mar 6;12(2):e81. doi: 10.1192/bjo.2025.10949 (PMC13107326; doi:10.1192/bjo.2025.10949)
Supplement: Lal et al. supplementary material 2 — Lal et al. supplementary material [file S2056472425109496sup002.pdf]

Supplementary tables.

Table S1 Joint effect of smoking status (all tobacco product smokers vs ex-smokers) and psychological distress (measured by K10 and SF 36 MHD) on expenditure items (AU$)

	K10 scales Contrast (95 % CI)					SF-36 MHD tertiles Contrast (95 % CI)			
	Low	Moderate	High	Very high	Joint p-value	Tertile 3	Tertile 2	Tertile 1 (worst mental health)	Joint p-value
Alcohol	**444.1 ***** (228.3, 660.0)	**494.7 **** (178.2, 811.3)	**361.0 *** (68.0, 654.1)	-48.9 (-428.6, 330.9)	<0.001	**221.7 *** (48.0, 395.4)	**243.2 **** (92.9, 393.4)	**286.3 ***** (136.0, 436.6)	<0.001
Clothing	**-341.7 ***** (-474.4, -209.0)	-255.8 (-735.6, 224.3)	-148.9 (-398.9, 101.0)	24.9 (-426.1, 475.9)	<0.001	**-295.4 ***** (-440.5, -150.7)	**-265.7 ***** (-415.7, -115.6)	-116.8 (-365.0, 131.4)	<0.001
Education	**-592.7 ***** (-870.3, -315.2)	**-476.3 **** (-822.6, -130.0)	**-566.3 **** (-909.2, -203.3)	**-464.2 *** (-844.8, -83.7)	<0.001	**-337.6 **** (-564.2, -111.0)	**-440.8 ***** (-627.1, -254.6)	**-507.2 ***** (-699.2, -315.2)	<0.001
Fuel	-247.5 (-509.0, 14.2)	-42.8 (-337.7, 252.1)	-73.0 (-870.2, 724.2)	-590.8 (-1222.4, 40.9)	<0.05	-199.9 (-401.3, 1.5)	**-195.0 *** (-388.8, -1.2)	-149.5 (-349.7, 50.6)	0.105
General insurance	**-489.5 ***** (-634.9, -344.1)	**-544.7 ***** (-741.3, -348.1)	**-347.9 **** (-603.5, -92.3)	**-358.8 *** (-654.5, -63.2)	<0.001	**-380.8 ***** (-508.7, -252.9)	**-412.7 ***** (-526.8, -298.6)	**-326.5 ***** (-438.8, -214.2)	<0.001
Groceries	-334.3 (-748.2, 79.6)	-257.1 (-850.2, 336.0)	-630.0 (-1341.4, 81.4)	-642.7 (-1436.0, 150.7)	0.118	-3.1 *** (-165.0, 158.9)	-17.2 (-143.5, 107.9)	-7.1 (-138.6, 124.4)	0.994
Health practitioner	**-345.6 ***** (-460.6, -230.5)	**-442.0 ***** (-642.4, -241.7)	-374.1 (-831.7, 83.5)	**-561.4 **** (-939.6, -183.1)	<0.001	**-906.1 ***** (-1271.9, -540.3)	-219.4 (-581.5, 142.7)	-275.2 (-570.6, 20.1)	<0.001
Internet	36.1 (-139.4, 211.5)	-69.6 (-267.2, 128.1)	**-286.7 **** (-500.4, -73.1)	-171.1 (-441.2, 99.1)	0.073	-3.1 (-165.0, 158.9)	-17.7 (-143.4, 107.9)	-7.1 (-138.6, 124.4)	0.994
Meals out	**-402.4 ***** (-629.9, -174.9)	**-635.8 ***** (-903.9, -367.8)	-330.8 (-682.1, 20.5)	**-677.0 ***** (-1078.4, -275.7	<0.001	**-476.4 ***** (-672.3, -280.9)	**-267.0 **** (-469.4, -64.5)	**-360.3 ***** (-499.6, -221.1)	<0.001
Medicines	**-125.4 ***** (-172.4, -78.4)	**-166.1 ***** (-228.0, -104.2)	-43.1 (-155.9, 69.5)	-65.8 (-201.7, 70.1)	<0.001	**-77.0 **** (-129.1, -24.9)	-42.1 (-87.6, 3.4)	-34.1 (-78.9, 10.8)	<0.05
Public transport	-49.5 (-121.4, 22.4)	-74.1 (-202.1, 54.0)	-79.0 (-198.9, 41.0)	-16.6 (-156.6, 123.3)	0.333	-61.0 (-134.6, 12.6)	**-92.4 ***** (-147.2, -37.6)	**-59.5 *** (-114.8, -4.2)	<0.01
Rent	**81.4 ***** (36.6, 126.1)	**91.0 ***** (37.2, 144.9)	5.4 (-55.7, 66.6)	-33.8 (-129.0, 61.5)	<0.001	**58.9 ***** (24.8, 93.0)	**44.3 *** (9.0, 79.6)	**36.5 **** (9.0, 64.0)	<0.01
Utilities	-10.9 (-147.3, 125.4)	-7.5 (-208.8, 193.7)	-74.7 (-287.1, 137.7)	-204.9 (-473.8, 64.0)	0.631	-73.5 (-92.7, 239.8)	-1.8 (-120.1, 116.5)	-41.7 (-146.3, 63.0)	0.516

Notes: Confidence interval; K10: Kessler Psychological Distress Scale; SF-36 MHD: Mental health domain of 36-Item short form health survey. Bold font indicates significance; * *p* < 0.05, **p <0.01, *** p < 0.001.

.

Table S2 Mean Expenditure ($) by K10 strata in T1 for cigarette-only smokers and ex-smokers.

K10 scales		Low ($, SD)	Moderate ($, SD)	High ($, SD)	Very high ($, SD)
Alcohol	Smokers	2288.3 (2905.5)	2577.7 (3576.2)	1803.8 (2507.3)	1436.2 (2643.2)
	Ex-smoker	1840.5 (2161.4)	1833.9 (2294.7)	1424.0 (1806.7)	1087.9 (1758.6)
Clothing	Smokers	1235.3 (1448.1)	1245.26 (1200.7)	1167.70 (1175.9)	1063.66 (1256.9)
	Ex-smoker	1571.0 (1782.6)	1621.02(3329.2)	1556.67 (2032.4)	1179.92 (1351.9)
Education	Smokers	738.6 (3080.3)	1255.64 (4140.2)	296.74 (809.6)	132.49 (370.1)
	Ex-smoker	1610.6 (6148.7)	1262.70 (3733.3)	1497.97 (5402.8)	701.01(1665.4)
Fuel	Smokers	2139.7 (1779.7)	2529.92 (2284.4)	2551.67 (7597.4)	1527.07(1764.3)
	Ex-smoker	2467.5 (2816.9)	2146.17(1855.3)	2269.12 (1803.1)	1867.31(2155.6)
General insurance	Smokers	1687.6 (1469.5)	1443.11 (1514.9)	1500.34 (3062.2)	867.47 (1090.8)
	Ex-smoker	2331.4 (1965.1)	2143.79 (1783.6)	1867.27 (1625.9)	1415.74 (1331.5)
Groceries	Smokers	9756.8 (5121.2)	10068.41(4837.5)	9237.67 (5459.2)	8701.98 (7302.8)
	Ex-smoker	10546.2 (6261.4)	10558.36 (5555.7)	10656.84 (7012.8)	9200.45(5139.9)
Health practitioner	Smokers	712.6 (1199.1)	879.30 (1774.2)	1183.92 (5807.5)	366.08 (810.7)
	Ex-smoker	1306.6 (2384.1)	1393.61 (2485.8)	1963.36 (8511.9)	721.98(1347.0)
Internet	Smokers	1429.9 (1610.0)	1498.48 (1471.4)	1202.01 (928.6)	1400.99 (2363.2)
	Ex-smoker	1423.8 (1363.9)	1527.20 (3697.4)	1329.01(1362.5)	1491.73 (1463.4)
Meals out	Smokers	2704.2 (2923.6)	2444.97 (2245.1)	2424.51(3266.6)	1454.30 (2160.4)
	Ex-smoker	3039.9 (3278.1)	3194.14 (4638.5)	2564.26 (2553.1)	2384.77(2166.5)
Medicines	Smokers	418.4 (633.3)	407.60 (529.5)	477.24 (955.3)	396.74 (503.0)
	Ex-smoker	585.4 (1447.0)	633.96 (820.8)	587.96 (643.2)	612.48 (807.6)
Public transport	Smokers	344.2 (874.0)	437.16 (924.5)	239.84 (607.6)	484.54 (972.9)
	Ex-smoker	333.1 (930.5)	505.24 (2963.2)	533.92 (1236.7)	194.55 (513.5)
Rent	Smokers	401.6 (683.9)	514.49 (700.8)	380.01 (569.9)	511.64 (590.7)
	Ex-smoker	188.5 (603.7)	306.07 (623.4)	327.99 (638.9)	480.81 (660.1)
Utilities	Smokers	1960.4 (1533.4)	2021.92 (1662.3)	1904.29 (1421.3)	1246.04 (1338.1)
	Ex-smoker	2156.4 (3285.9)	2184.16 (1787.2)	2070.09 (1520.1)	1877.39 (1773.2)

Notes: SD Standard Deviation. K10: Kessler Psychological Distress Scale
